# Comparative study on the mutational profile of adenocarcinoma and squamous cell carcinoma predominant histologic subtypes in Chinese non‐small cell lung cancer patients

**DOI:** 10.1111/1759-7714.13208

**Published:** 2019-11-06

**Authors:** Ying Ding, Lihua Zhang, Lingchuan Guo, Chunyan Wu, Jianhua Zhou, Yongchun Zhou, Jie Ma, Xiao Li, Pan Ji, Ming Wang, Weidong Zhu, Chenxi Shi, Sanen Li, Wei Wu, Wei Zhu, Desheng Xiao, Chunyan Fu, Qiuyan He, Rui Sun, Xinru Mao, Analyn Lizaso, Bing Li, Han Han‐Zhang, Zhihong Zhang

**Affiliations:** ^1^ Department of Pathology The First Affiliated Hospital of Nanjing Medical University Nanjing China; ^2^ Department of Pathology Southeast University, Zhongda Hospital Nanjing China; ^3^ Department of Pathology The First Affiliated Hospital of Soochow University Suzhou China; ^4^ Department of Pathology Shanghai Pulmonary Hospital, Tongji University Shanghai China; ^5^ Department of Pathology Xiangya Hospital, Central South University Changsha China; ^6^ Molecular Diagnostic Center, Yunnan Cancer Center, Yunnan Cancer Hospital Kunming China; ^7^ Department of Molecular Pathology The Affiliated Cancer Hospital of Zhengzhou University Zhengzhou China; ^8^ Burning Rock Biotech Guangzhou China

**Keywords:** Adenocarcinoma, histological subtyping, molecular profiling, non‐small cell lung cancer, squamous cell carcinoma

## Abstract

**Background:**

Distinction in the mutational profile between the common histological types, lung adenocarcinoma (LUAD) and squamous cell lung carcinoma (LUSC) has been well‐established. However, comprehensive mutation profiles of the predominant histological subtypes within LUAD and LUSC remains elusive.

**Methods:**

We analyzed the mutational profile of 318 Chinese NSCLC patients of adenocarcinoma and squamous cell carcinoma predominant subtypes from seven hospitals using capture‐based ultra‐deep sequencing of 68 lung cancer‐related genes.

**Results:**

Of the 318 NSCLC patients, 215 were diagnosed with LUAD and 103 with LUSC. Adenocarcinoma in situ and acinar adenocarcinoma were the most predominant subtypes of LUAD. On the other hand, keratinizing squamous cell carcinoma was the most predominant subtype of LUSC. Among the LUAD subtypes, *EGFR* sensitizing mutations were most prevalent in the invasive lepidic subtype. More than half of the patients with preinvasive adenocarcinoma in situ, minimally invasive, acinar, micropapillary and papillary subtypes were also *EGFR*‐mutants. Patients with colloidal, invasive mucinous, and fetal subtypes had the least number of *EGFR* mutations. Moreover, *KRAS* mutations were prevalent in patients with invasive mucinous, colloid, enteric and solid subtypes. A total of 90% of the LUSC patients harbor mutations in *TP53*, wherein all patients except five with nonkeratinizing were *TP53* mutants. *PIK3CA* amplifications were most prevalent in keratinizing, followed by basaloid and nonkeratinizing subtypes.

**Conclusion:**

These data suggest that the mutational profiles among the predominant histological subtypes were very distinct, which provided a reliable tool to improve treatment decisions.

## Keypoints

- Mutation profiling can aid in distinguishing between the histological subtypes to improve treatment decisions.
- Lung adenocarcinoma and squamous cell lung carcinoma are molecularly distinct.
- The most predominant lung adenocarcinoma subtypes were adenocarcinoma in situ and acinar adenocarcinoma. The most predominant squamous cell lung carcinoma subtype was keratinizing squamous cell carcinoma.

## Introduction

Lung cancer is the leading cause of cancer‐related death in China and around the world.^1^, ^2^ Non‐small cell lung cancer (NSCLC) accounts for about 85% of lung cancer cases diagnosed, with two major histological types: adenocarcinoma (LUAD) and squamous cell carcinoma (LUSC) accounting for nearly 50% and 30% of NSCLC, respectively.^3^ Historically, the histological classification of NSCLC had not been a major determining factor in treatment guidance.^4^ It is only in the past decade that it has become apparent that LUAD and LUSC have distinctive mutation profiles, responsible for their divergent responses to targeted therapies. Development of targeted therapies such as EGFR‐TKIs has revolutionized the management of *EGFR*‐mutant LUAD patients.^5^, ^6^ Furthermore, pemetrexed^7^ and bevacizumab^8^ were approved for nonsquamous NSCLC. On the other hand, nivolumab has recently been approved by the U.S. Food and Drug Administration for metastatic LUSC patients.^9^ Due to the development of therapeutic agents approved only for particular histological types, the need for histopathological classification grew significantly over the years.

Conventionally, histological classification of NSCLC primarily relied on morphology using light microscopy with hematoxylin‐eosin and mucin stains. However, certain cytology samples obtained from small biopsies are morphologically indistinguishable such as poorly differentiated adenocarcinoma and squamous cell.^10^ Immunohistochemistry (IHC) markers were introduced in the diagnosis of NSCLC to improve accuracy and reproducibility of histological classification and are now widely used in the subtyping of NSCLC. In line with the growing need for histologic classification of NSCLC, the World Health Organization (WHO) recently revised the guidelines for the classification of lung tumors. Some of the amendments included the emphasis of histology to personalized medicine and modification of the histologic criteria and classifications for both LUAD and LUSC subtypes following the recommendations from the Association for the Study of Lung Cancer, American Thoracic Society and European Respiratory Society (ASLC/ATS/ERS).^4^ The reclassification in the LUAD and LUSC subtypes were according to the predominant morphologic pattern as well as the general pattern of invasion. LUAD with predominantly lepidic, nonmucinous pattern is characterized as preinvasive adenocarcinoma in situ, minimally invasive adenocarcinoma, or invasive adenocarcinoma with lepidic component depending on the invasion pattern; while other invasive LUAD with identifiable patterns are classified as invasive mucinous, colloid, fetal, enteric, acinar, papillary, micropapillary, and solid subtypes.^4^ On the other hand, LUSC is further classified as preinvasive squamous cell carcinoma in situ, and invasive squamous cell carcinoma as keratinizing, nonkeratinizing and basaloid subtypes.^4^

Each cancer histological type and subtype arose from multiple risk factors including genetic and environmental and thus has its own unique genetic mutational profile. Molecular profiling of the individual tumor's genome facilitates the understanding of the distinct molecular mechanism that regulates cancer progression and the discovery of potential therapeutic targets. Due to the advancements in molecular profiling technologies, the mutation profiles of LUAD and LUSC have been well elucidated.^11^, ^12^, ^13^ However, the molecular distinction between each specific histological subtypes within LUAD and LUSC is just beginning to be understood. Among LUAD subtypes, *EGFR* mutations are more prevalent in lepidic (formerly termed as bronchioalveolar) tumors, while *KRAS* mutations are more common invasive LUAD subtypes, particularly invasive mucinous and solid subtypes.^11^, ^13^, ^14^, ^15^, ^16^, ^17^, ^18^, ^19^ On the other hand, limited information is available on the molecular distinction in LUSC subtypes.^13^

In this multi‐center comparative study, we aimed to characterize the mutational profiles of the major histological subtypes of adenocarcinoma and squamous cell carcinoma in the Chinese NSCLC patients. This study emphasizes the need for mutational profiling in all NSCLC patients to identify actionable mutations amenable to targeted therapy.

## Methods

### Patient enrollment

This cohort included 318 Chinese stage I–IV NSCLC patients with either adenocarcinoma or squamous cell types from seven hospitals. All patients underwent complete tumor staging according to the seventh edition tumor, node, and metastasis (TNM) criteria of NSCLC.^20^ Lung cancer histology was classified according to the predominant subtype following the 2015 World Health Organization (WHO) histopathology classification.^4^ The patients' clinical data, including demographic information, smoking status and cancer histological subtype, were reviewed. Tumor samples were obtained either by surgical or needle biopsy procedures and sequenced for mutational analysis. The study had been approved by the relevant regulatory and independent ethics committees or institutional review boards of all the participating hospitals. Written informed consent was obtained from each patient for the use of their tissue samples.

### Tissue DNA extraction

DNA was extracted from formalin‐fixed, paraffin‐embedded (FFPE) tumor tissues using QIAamp DNA FFPE tissue kit (Qiagen, Hilden, Germany) according to the manufacturer's instructions.

### Capture‐based targeted DNA sequencing

A minimum of 50 ng of DNA is required for NGS library construction. Tissue DNA was sheared using Covaris M220, followed by end repair, phosphorylation, and adaptor ligation. Fragments of size 200–400 bp were selected by bead (Agencourt AMPure XP Kit, Beckman Coulter, Brea, CA, USA), followed by hybridization with capture probes baits, hybrid selection with magnetic beads and PCR amplification. Lung Core panel from Burning Rock Biotech (Guangzhou, China) consisting of 68 lung cancer‐related genes spanning 345 kb of the human genome was used. The quality and the size of the fragments were assessed using a Qubit 2.0 Fluorimeter with the dsDNA high sensitivity assay kit (Life Technologies, Carlsbad, CA, USA). Indexed samples were sequenced on Nextseq500 (Illumina, Inc., Madison, WI, USA) with paired‐end reads.

### Sequence data analysis

Sequence data were mapped to the reference human genome (hg19) using Burrows‐Wheeler aligner v.0.7.10. Local alignment optimization, variant calling and annotation were performed using Genome Analysis Tool Kit v.3.2, and VarScan. Variants were filtered using the VarScan fpfilter pipeline, loci with depth less than 100 were filtered out. Base calling in tissue samples required at least five and eight supporting reads for small insertion‐deletions (INDELs) and single nucleotide variants (SNVs), respectively. INDELs and SNVs with population frequency over 0.1% in the ExAC, 1000 Genomes, dbSNP or ESP6500SI‐V2 databases were grouped as SNP and excluded from further analysis. Remaining variants were annotated with ANNOVAR and SnpEff v.3.6. Analysis of DNA translocation was performed using Factera v.1.4.3.

### Statistical analysis

All the data were analyzed using R software (R version 3.4.0; R: The R Foundation for Statistical Computing, Vienna, Austria). Significance between the groups was calculated using Fisher's exact test with *P* < 0.05 considered as statistically significant. *P*‐values were adjusted for variables such as age, gender, smoking history, and pathological stage when applicable.

## Results

### Patient characteristics

**Figure 1:**
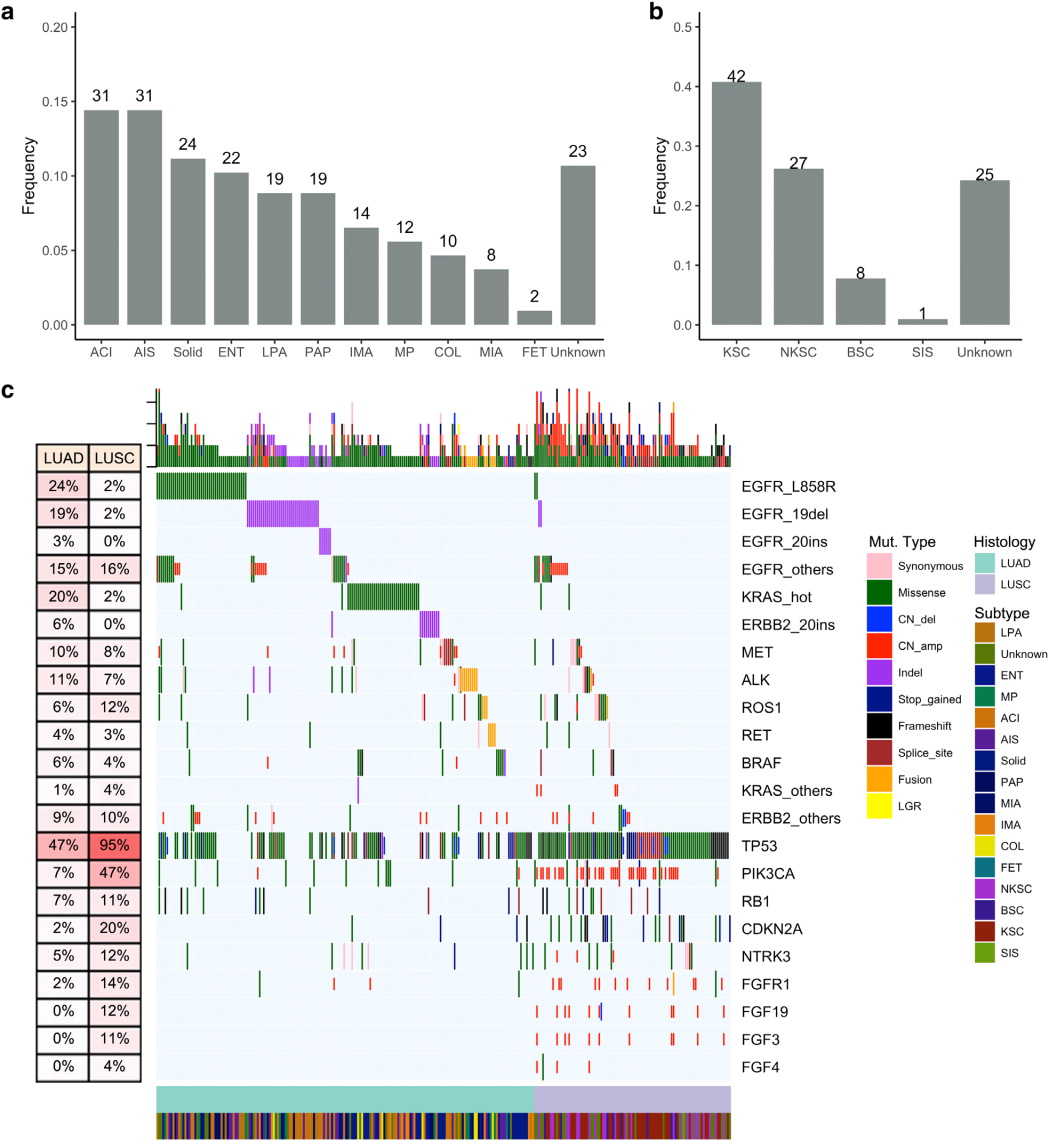
Histological subtype distribution and mutational profile of the lung cancer patients in the cohort. Distribution of (**a**) LUAD and (**b**) LUSC patients according to their histological subtypes. X‐axis denotes the histological subtype, Y‐axis, patient frequency. (**c**) Mutational spectrum of the patients grouped according to histological subtype. Each column represents a patient and each row represents a gene. Table on the left represents the mutation rate of each gene that corresponds to either LUAD or LUSC histological type. Top plot represents the overall number of mutations a patient carried. Different colors denote different types of mutation. ACI, acinar adenocarcinoma; AIS, adenocarcinoma in situ; BSC, basaloid squamous cell carcinoma; COL, colloid adenocarcinoma; ENT, enteric adenocarcinoma; FET, fetal adenocarcinoma; IMA, invasive mucinous adenocarcinoma; KSC, keratinizing squamous cell carcinoma; LPA, lepidic adenocarcinoma; LUAD, adenocarcinoma; LUSC, lung squamous cell carcinoma; MIA, minimally invasive adenocarcinoma; MP, micropapillary adenocarcinoma; NKSC, nonkeratinizing squamous cell carcinoma; PAP, papillary adenocarcinoma; SIS, squamous cell carcinoma in situ; Solid, solid adenocarcinoma.

Our cohort consisted of 318 Chinese stage I‐IV NSCLC patients diagnosed with either lung adenocarcinoma (LUAD) or lung squamous cell carcinoma (LUSC) from seven hospitals. Lung cancer histological subtypes other than LUAD and LUSC were excluded from the study cohort. Patient demographics and clinical characteristics are summarized in Table 1. Figure 1 illustrates the distribution of LUAD and LUSC patients according to their histological subtypes. Of the 215 LUAD patients, the most predominant LUAD subtypes were adenocarcinoma in situ and acinar subtypes with 14.4% (31/215) each. Other LUAD subtypes included solid (24/215, 11.2%), enteric (22/215, 10.2%), lepidic (19/215, 8.8%), papillary (19/215, 8.8%), invasive mucinous (14, 6.5%), micropapillary (12/215, 5.6%), colloid (10/215, 4.7%), minimally invasive (8/215, 3.7%) and fetal (2/215, 1%). There were 23 (10.7%) patients with unclassifiable LUAD (Fig 1a). On the other hand, among the 103 LUSC patients, keratinizing subtype (40.8%, 42/103) was the most predominant. Other LUSC subtypes included non‐keratinizing (26/103, 26.2%), basaloid (8/103, 8%), and squamous cell carcinoma in situ (1/103, 1%). There were 25 (24.3%) patients with unclassifiable LUSC (Fig 1b). The pathologic stage distribution of the 215 LUAD and 103 LUSC patients according to predominant histological subtypes were summarized in Tables S1 and S2, respectively.

**Table 1:** Patient characteristics

Clinical characteristics	Total *n* = 318	LUAD *n* = 215	LUSC *n* = 103
Age (years)			
Median, (range)	61, (29–84)	61, (29–81)	61, (36–84)
Gender			
Male	201 (63.2%)	106 (49.3%)	95 (92.2%)
Female	117 (36.8%)	109 (50.7%)	8 (7.8%)
Smoking history			
Smoker	92 (28.9%)	40 (18.6%)	52 (50.5%)
Non‐smoker	166 (52.2%)	143 (66.5%)	23 (22.3%)
Unknown	60 (18.9%)	32 (14.9%)	28 (27.2%)
Clinical stage			
Stage IA	65 (20.4%)	50 (23.3%)	15 (14.6%)
Stage IB	32 (10.1%)	22 (10.2%)	10 (9.7%)
Stage IIA	15 (4.7%)	6 (2.8%)	9 (8.7%)
Stage IIB	15 (4.7%)	4 (1.9%)	11 (10.7%)
Stage IIIA	32 (10.1%)	24 (11.2%)	8 (7.8%)
Stage IIIB	6 (1.9%)	3 (1.4%)	3 (2.9%)
Stage IV	16 (5.0%)	13 (6.0%)	3 (2.9%)
NA	137 (43.1%)	93 (43.3%)	44 (42.7%)

### Comparison of the mutational profile of LUAD and LUSC patients

Of 297 patients, 199 (92.6%) were LUAD and 94 (91.3%) were LUSC. A total of 31 patients (16 LUAD and 15 LUSC) had no mutation detected from our panel.

From the somatic mutational profile of the patients, a distinct pattern between LUAD and LUSC was observed. Mutations in the oncogenic drivers were more prevalent in LUAD than LUSC patients (*P* < 0.001), with 77.2% (166/215) of the LUAD patients harboring alteration in oncogenic driver mutations. Oncogenic drivers included *EGFR* L858R and exon 19 deletion (19del), *KRAS* G12, G13 and Q61, BRAF V600E, *MET* exon14 skipping and amplification, *ERBB2* exon 20 insertion, *ALK* fusions, *RET* fusions, *ROS1* fusions and *NRG1* fusions. Among them, 37.7% (81/215) of the patients carried *EGFR* L858R and 19del mutations. Mutual exclusivity was observed among driver mutations in LUAD. However, a rare co‐occurrence of *EGFR* L858R and *KRAS* Q61H was found in a LUAD patient. Moreover, we also observed concurrent mutations of *EGFR* L858R and *ERBB2* S310F in two adenocarcinoma patients; *EGFR* L858R and *ERBB2* amplification in three LUAD patients (Table S3). In addition to sensitizing mutations in *EGFR*, we found three rare *EGFR* mutations including *EGFR* G719A detected in a patient and *EGFR* S758I and L838V concurrently detected in a patient (Table S3). A total of 24 patients (11.2%, 24/215) had *EGFR* compound mutation. *KRAS* mutations, including G12, G13, Q61, were detected in 17.7% (38/215) of patients. Among the *BRAF* mutations detected in 5.6% (12/215) of patients, no BRAF V600E was found. A BRAF rare disruptive in‐frame insertion R506_K507insVLR was detected in a LUAD patient (Table S4). Conversely, mutations in *ALK*, *RET* and *ROS1*, including fusions and other types of mutations, were detected in 25 (11.6%), 8 (3.7%) and 12 (5.6%) LUAD patients, respectively. A majority (77.8%, 7/9) of the patients with *ALK* fusion had *EML4* as the fusion partner. Of these nine patients, four had single *EML4‐ALK* fusion, while three patients had *EML4‐ALK* and concurrent *ALK* fusion with previously unreported partners, such as *EXOC6B*, *TTN*, *ACVR1*, and *TACR1*. The two remaining patients had other unreported *ALK* fusion partners, including *ERBB4* found in a patient and both *PRR20A* and *RHOB* detected in another patient. A summary of the *ALK* fusions detected in our cohort was listed in Table S4. Moreover, a patient with a driver *CCDC6‐RET* fusion also had a concurrent, previously unreported *RET* fusion with *CCSER2*. In addition, *CD74‐NRG1* fusions were also detected in three LUAD patients. Rare mutations and concurrent mutations in oncogenic genes were summarized in Table S3.

In LUSC, only 10 (9.7%, 10/103) patients carried alterations in oncogenic drivers. No mutual exclusivity was observed among driver mutations. Interestingly, four and one LUSC patients carried *EGFR* sensitizing and *KRAS* mutations, respectively. Such mutations were believed to occur exclusively in LUAD. On the other hand, *BRAF* mutations were found in four (3.9%, 4/103) LUSC patients; however, no *BRAF* V600E mutations were detected. Conversely, *EML4‐ALK* and *CD74‐ROS1* fusions were found in a patient each; while no *RET* fusion was found in LUSC patients.

On the other hand, mutations in *TP53* (*P* < 0.001) and gene amplifications in particular genes (*P* < 0.001) were more predominant in LUSC. *TP53* mutations were detected in almost all of the LUSC patients (90%, 93/103) but only 41% (88/215) of LUAD patients. In addition, LUSC patients had significantly more mutations in *CDKN2* (20/103, *P* < 0.001), and amplifications in *CCND1* (14/103, *P* < 0.001), *PIK3CA* (40/103, *P* < 0.001) and *FGFR1* (13/103, *P* < 0.001) than LUAD patients. Amplifications in *FGF19* (10.7%, 11/103), *FGF3* (10.7%, 11/103) and *FGF4* (2.9%, 3/103) were only found in LUSC patients in our cohort (Fig 1c). Interestingly, a previously unreported *FGFR1* fusion with *KCNU1* (*KCNU1* intergenic: *FGFR1* F4) was detected in a patient also harboring concurrent *FGFR1* amplification and NTRK1 Q558X stop gain mutation. Moreover, *PIK3CA* mutation types between LUAD and LUSC were significantly different, with missense mutations more prevalent in LUAD patients, while amplifications were largely found in LUSC patients (*P* < 0.001, Fig 1c). These data indicate that LUAD and LUSC have very distinct mutational characteristics (*P* < 0.001).

### Mutational profile of major subtypes of lung adenocarcinoma patients (LUAD)

**Figure 2:**
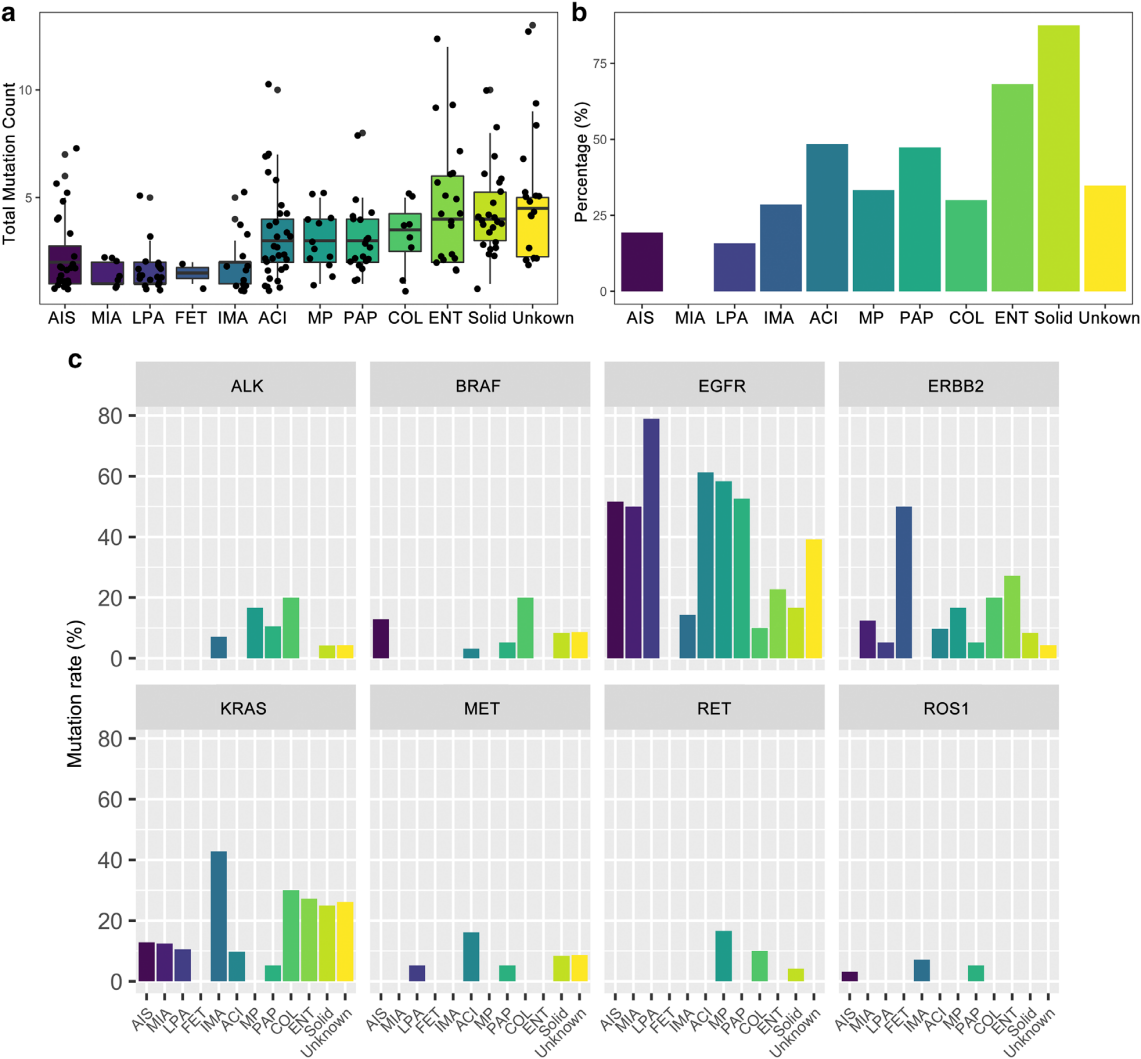
Mutational profile of LUAD patients based on predominant histological subtype. (**a**) Total mutation count (including small nucleotide variations, insertion‐deletions, copy number variations and fusions) in LUAD patients. X‐axis denotes the LUAD subtypes. Y‐axis denotes the total mutation count per patient. Each dot represents a patient. (b) Mutational profile in oncogenic gene drivers including *ALK* fusion, *BRAF* mutations, *EGFR* mutations and amplifications, *ERBB2* exon20 insertion and amplifications, *KRAS* G12, G13 and Q61 mutations, *MET* exon14 skipping and amplifications, *RET* fusion, *ROS1* fusion in LUAD patients. (**c**) Mutation rate in *TP53* in LUAD patients. X‐axis denotes the LUAD subtype. Y‐axis denotes the mutation rate, calculated as a percentage of the ratio of patients positive for the gene mutation against the total number of patients in the particular subtype group. ACI, acinar adenocarcinoma (*n* = 31); AIS, adenocarcinoma in situ (*n* = 31); COL, colloid adenocarcinoma (*n* = 10); ENT, enteric adenocarcinoma (*n* = 22); FET, fetal adenocarcinoma (*n* = 2); IMA, invasive mucinous adenocarcinoma (*n* = 14); LPA, lepidic adenocarcinoma (*n* = 19); MIA, minimally invasive adenocarcinoma (*n* = 8); MP, micropapillary adenocarcinoma (*n* = 12); PAP, papillary adenocarcinoma (*n* = 19); Solid, solid adenocarcinoma (*n* = 24); Unknown, unclassified LUAD subtype (*n* = 23).

Our data revealed oncogenic mutation detection rate of 80.6% (25/31), 75% (6/8) and 89.5% (137/153) for preinvasive, minimally‐invasive and invasive subtypes, respectively. Patients with invasive subtypes (ie, acinar, colloid, enteric, fetal, lepidic, micropapillary, invasive mucinous, papillary, and solid adenocarcinomas) harbored more total mutations than preinvasive (ie, adenocarcinoma in situ) and minimally‐invasive subtypes (*P* < 0.001, Fig 2a).

In general, *EGFR* sensitizing mutations were predominant in LUAD (*P* < 0.001) with relatively even distribution in preinvasive, minimally invasive and invasive LUAD subtypes (*P* = 0.515, Fig 2b). However, further analysis of the detailed subtypes revealed that among all the subtypes, invasive lepidic adenocarcinoma had the most *EGFR*‐mutant patients (*P* = 0.003), followed by acinar and micropapillary subtypes. Meanwhile, both patients with fetal subtype were wild‐type for *EGFR* (Fig 2b).

Among the patients with preinvasive adenocarcinoma in situ (AIS) subtype, *EGFR* mutations were detected in 52% (16/31) of the patients (Fig 2b). No alterations in *ALK*, *ERBB2*, *MET*, and *RET* were found in AIS patients (Fig 2b). Only an AIS patient harbored *ROS1* fusion. Meanwhile, *TP53* mutations were found in 19% (6/31) of the AIS patients (Fig 2c). Among the patients with no mutations detected from our panel, five were AIS patients.

In patients with minimally invasive adenocarcinoma subtype (MIA), the only oncogenic driver mutations detected were *EGFR*, *ERBB2* and *KRAS* (Fig 2b) wherein half (4/8) of the MIA patients were *EGFR* mutants. *ERBB2* and *KRAS* were found in a patient each. No gene alterations were detected in *ALK*, *BRAF*, *MET*, *RET*, *ROS1* and *TP53* in MIA patients (Fig 2b). An MIA patient was negative for mutations in our gene panel.

Among all the patients in the cohort, patients with adenocarcinoma of the invasive type generally had alterations in all oncogenic driver genes. In particular, gene alterations in *ALK*, *MET* and *RET* were only found in these patients (Fig 2b). *TP53* mutations were also more prevalent in patients with invasive adenocarcinoma (48%, 74/153, Fig 2c).

Not only among invasive subtypes but among all the LUAD subtypes, lepidic adenocarcinoma subtype (LPA) had the most *EGFR* mutations, with 78.9% (15/19) *EGFR* positive patients (*P* = 0.003, Fig 2b) with the majority being sensitizing mutations. No *ALK*, *BRAF*, *RET* and *ROS1* alterations were detected in LPA patients (Fig 2b). Mutations in *ERBB2*, *MET* and *KRAS* were found in 1, 1, and 2 LPA patients (Fig 2b); while mutations in *TP53* were detected in three LPA patients (Fig 2c).

Patients with acinar subtype (ACI) had mutations in all oncogenic driver genes (Fig 2b). *EGFR* mutations were found in 61% (19/31) of these patients (Fig 2b). *TP53* mutations were detected in half of the ACI patients (15/31) (Fig 2c). No fusions in *ALK*, *RET* and *ROS1* were detected in ACI patients.

Solid adenocarcinoma patients had the most *TP53* mutations (87.5%, 21/24) among all LUAD subtypes (Fig 2c). Mutations in all oncogenic drivers were detected in these patients except *ROS1* fusion (Fig 2b). *KRAS* mutations were found in 25% (6/24) of the patients with solid subtype; while *EGFR* mutations were only found in 16.7% (4/24) of the patients with solid subtype.

Patients with enteric adenocarcinoma (ENT) did not have mutations in *ALK*, *BRAF*, *MET*, *RET* and *ROS1*. The only mutations found in ENT patients were *EGFR* (22.7%, 5/22), *ERBB2* (27.3%, 6/22) and *KRAS* (27.3%, 6/22) (Fig 2b). Of the 22 ENT patients, 68% (15/22) had *TP53* mutations (Fig 2c). Two ENT patients had no mutation detected from our panel.

Except for *RET* fusion, patients with papillary subtype (PAP) had mutations in all oncogenic driver genes with 52.6% (10/19) of them positive for *EGFR* sensitizing mutations (Fig 2b). In addition, half of the PAP patients (9/19) had *TP53* mutation (Fig 2c).

Patients with invasive mucinous adenocarcinoma subtype (IMA) patients had the most prevalent *KRAS* mutations, with 40% (6/14) *KRAS*‐positive IMA patients (Fig 2b). Instead of sensitizing mutations, only *EGFR* amplifications were found in two IMA patients (Fig 2b). No *BRAF*, *ERBB2*, *MET* and *RET* alterations were detected in any of these patients (Fig 2b). A patient with IMA was negative for mutations in our gene panel.

Among the 12 patients with micropapillary adenocarcinoma subtype (MP), 58.3% (7/12) were positive for *EGFR* sensitizing mutations (Fig 2b). Oncogenic driver mutations in MP patients were only detected in *EGFR* (7/12), *ALK* (2/12), *ERBB2* (2/12) and *RET* (2/12) (Fig 2b). The four MP patients with *TP53* mutations also coharbored *EGFR* sensitizing mutations (Figure S1).

Among the 10 colloid adenocarcinoma subtype (COL) patients, 30% (3/10) were positive for *KRAS* mutations (Fig 2b). Interestingly, two *KRAS*‐positive patients also had concomitant *BRAF* mutations (Fig 2b). Moreover, two patients had *ALK* fusion; one patient had *ERBB2* amplification and one had both EGFR 19del and *ERBB2* amplification (Fig 2b). Two patients with COL were negative for mutations in our gene panel.

Apart from *ERBB2* amplification and *KRAS* mutation in each of the two fetal adenocarcinoma (FET) patients in the cohort, no other gene alterations in any oncogenic driver and *TP53* were detected (Fig 2b,c).

### Mutational profile of squamous cell lung carcinoma patients (LUSC) according to subtype

**Figure 3:**
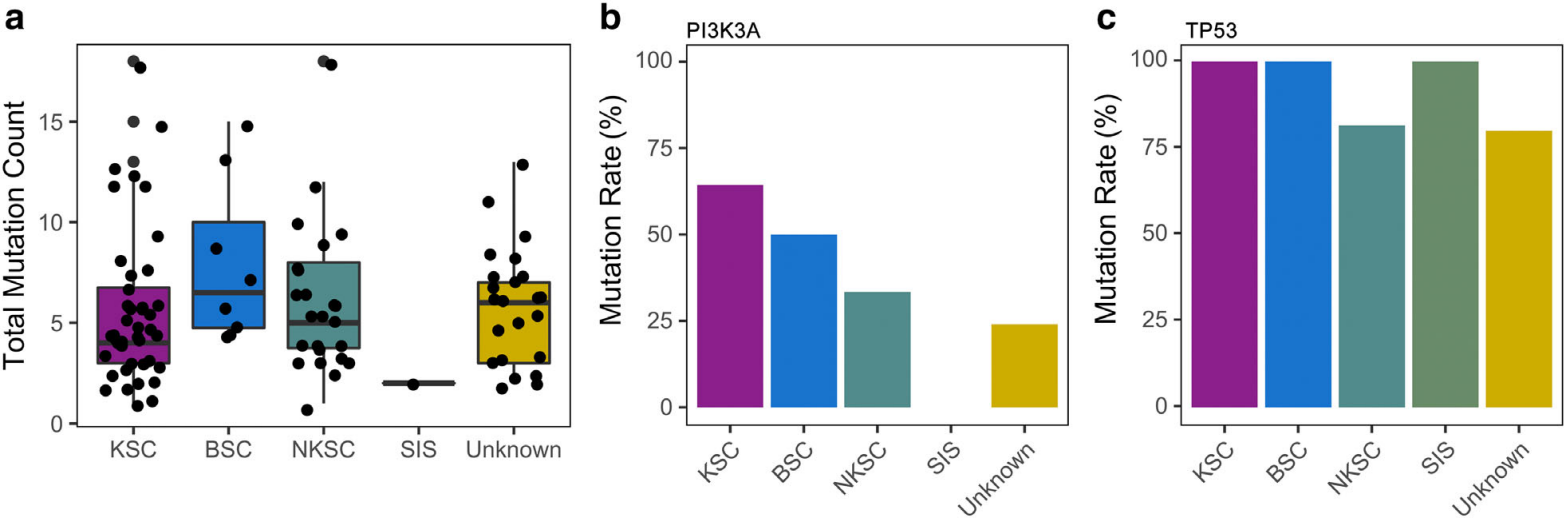
Mutational profile of LUSC patients based on predominant histologic subtype. (**a**) Total mutation count (including small nucleotide variations, insertion‐deletions, copy number variations and fusions) of LUSC patients. X‐axis denotes the LUSC subtypes. Y‐axis denotes the total mutation count per patient. Each dot represents a patient. Mutation rate in *PIK3CA* (**b**) and *TP53* (**c**) in LUSC patients. X‐axis denotes the LUSC subtype. Y‐axis denotes the mutation rate, calculated as the percentage of the ratio of patients positive for the gene mutation against the total number of patients in the particular subtype group. BSC, basaloid squamous cell carcinoma (*n* = 8); KSC, keratinizing squamous cell carcinoma (*n* = 42); NKSC, nonkeratinizing squamous cell carcinoma (*n* = 27); SIS, squamous cell carcinoma in situ (*n* = 1); Unknown, unclassified LUSC subtype (*n* = 25).

In contrast to LUAD, LUSC had a predominance of mutations in *TP53* and copy number variations in particular genes, while oncogenic driver mutations were very few (*P* < 0.001, Fig 1c, Figure S2). The mutation count among the LUSC histological subtypes were comparable, with the median basaloid squamous cell carcinoma (BSC) patients slightly higher but not statistically different from the other subtypes (*P* = 0.45, Fig 3a).

*PIK3CA* amplifications were most prevalent in keratinizing squamous cell carcinoma (KSC) patients (64.3%, 27/42, *P* = 0.015), followed by basaloid squamous cell carcinoma (BSC) (50%, 4/8) and nonkeratinizing squamous cell carcinoma (NKSC) (33.3%, 9/27). No *PIK3CA* mutation was found in the only squamous cell carcinoma in situ (SIS) patient (Fig 3b).

As expected for LUSC, *TP53* mutations were detected in almost all (90%, 93/103) of the LUSC patients, wherein all the patients with KSC (42/42), BSC (8/8) and SIS (1/1) were *TP53* mutants. Meanwhile, NKSC patients only had 81% (22/27) *TP53* mutants (Fig 3c). Of the five patients with wild‐type *TP53*, two of them had no mutation detected in any of the genes in our panel (Fig 3b, Figure S2). The other five wild‐type *TP53* patients had unclassified subtype (Fig 3c).

### The relationship between molecular and clinical features in LUAD and LUSC patients

In LUAD patients, *TP53* mutations were associated with older patients. The age of *TP53*‐positive LUAD patients ranged between 43 to 81 years with a median age of 62 years, while the age of wild‐type *TP53* patients ranged between 29 to 79 years with a median age of 59 years (adjusted *P* < 0.001, Figure S3). *KRAS* mutations were also found to be associated with smoking status (adjusted *P* = 0.039). There was no significant correlation in age, gender and genetic alterations among the LUSC patients.

## Discussion

The understanding and management of lung cancer has advanced significantly in the past decade. Even with the increasing importance of molecular testing to identify actionable mutations for targeted therapy, histopathological classification of cancer subtypes is still an essential component of clinical diagnosis and making optimal treatment decisions, particularly in patients with no actionable mutations.

To the best of our knowledge, our study is the first to use a unified strategy to compare the mutational profile of LUAD and LUSC predominant histological subtypes in Chinese NSCLC patients.

In our cohort of 215 LUAD NSCLC patients, adenocarcinoma in situ and acinar subtypes were the two most prevalent LUAD histological subtypes. This is in contrast to Caucasian histological prevalence where the top two subtypes were acinar followed by solid subtypes.^11^, ^21^ Since previous studies have only used traditional methods of molecular testing, existing literature on the genetic alterations in various histological subtypes are mostly limited to the detection rates of *EGFR* and *KRAS* mutations. *EGFR* mutations are detected in 10%–30% of LUAD patients; however, this prevalence increases to approximately 50% among Chinese LUAD patients.^13^, ^22^ Hence, we only considered the reports that included Chinese patients. A previous study reported that among Chinese LUAD patients, lepidic and micropapillary subtypes had the most *EGFR* mutations with approximately 70% *EGFR*‐mutant patients from each subtype, while solid subtype had the least number of *EGFR* mutant patients.^18^ Meanwhile, another study reported the *EGFR* mutation detection rates of 68.8%, 70.7%, 69.5%, 22.5%, 80.0%, and 25.0% in Chinese patients with lepidic, papillary, acinar, solid, micropapillary and mucinous subtypes, respectively, with no *EGFR* mutation detected in the case of fetal adenocarcinoma.^23^ In contrast, in our cohort, *EGFR* sensitizing mutations were generally more common in preinvasive and minimally invasive subtypes. Meanwhile, invasive subtypes such as acinar, micropapillary, and papillary also had a substantial number of *EGFR* mutant patients. However, the least number of *EGFR* sensitizing mutations were in patients of colloidal subtype (1/10), invasive mucinous (0/14) and fetal (0/2) subtypes. Conversely, *KRAS* mutations in our cohort were consistent with the reported prevalence.^17^ In our cohort, *KRAS* mutations were also more prevalent in invasive mucinous (6/14), colloid (3/10), enteric (4/22) and solid (6/24) subtypes.

Furthermore, we revealed distinct mutation profiles for Chinese LUAD and LUSC patients. In contrast to LUAD patients, the LUSC patients in our cohort had significantly more amplification events and *TP53* mutations. Similar to the findings of the TCGA^12^ and a study among 104 Korean LUSC patients by Kim *et al*.,^24^ significantly fewer *EGFR* and *KRAS* mutations were detected in Chinese LUSC than LUAD patients. Conversely, another mutational profiling study involving 157 Chinese LUSC patients reported mutation incidence of 56% for *TP53*, 8.9% for *CDKN2*, 8.9% for *PIK3CA*,^25^ and the incidence rates were significantly lower than those observed in our cohort. Studies involving larger cohorts are necessary to validate the incidence rates.

Interestingly, we have detected several rare mutations in known oncogenic genes including *EGFR* and *BRAF* and unreported fusion partners for *RET*, *FGFR1* and *ALK* in our cohort. With the increasing use of molecular profiling in the clinical setting, more novel mutations and fusion partner genes are being uncovered. However, the clinical significance of these rare mutations and novel fusion partners requires further studies. Our study is limited by its retrospective nature. Clinical data for some of the patients were incomplete which limits the analysis of clinical features and histological subtypes of the cohort. The limited availability of tissue samples also limits us to explore the clinical significance of rare mutations detected in the study. Future prospective multi‐center studies with a larger cohort are required to further explore the mutation profile of the different subtypes. It would be interesting to explore the stratification of molecular subtypes according to distinct mutation signature and their clinical responses to certain inhibitors.

In summary, this comparative study revealed the mutational distinction between LUAD and LUSC as well as their predominant histological subtypes in the Chinese population. Taking the inherent genetic heterogeneity among tumor subtypes, we further emphasize the need to include comprehensive mutational profiling in the standard management of lung cancer patients of all histological subtypes to understand the genetic landscape of the tumor and further inform clinical decisions.

## Disclosure

The authors declare no potential conflicts of interest.

## Supporting information


**Figure S1** Mutational spectrum of the LUAD patients. Each column represents a patient and each row represents a gene. Top plot represents the overall number of mutations a patient carried. Side bars represent the percentage of patients with a certain mutation. Different colors denote different types of mutation. Negative denotes the absence of any mutation. ACI, acinar adenocarcinoma; AIS, adenocarcinoma in situ; COL, colloid adenocarcinoma; ENT, enteric adenocarcinoma; FET, fetal adenocarcinoma; IMA, invasive mucinous adenocarcinoma; LPA, lepidic adenocarcinoma; MIA, minimally invasive adenocarcinoma; MP, micropapillary adenocarcinoma; PAP, papillary adenocarcinoma; Solid, solid adenocarcinoma; Unknown, LUAD with unclassified subtype.

**Figure S2** Mutational profile of LUSC patients according to histological subtypes. Each column represents a patient and each row represents a gene. Top plot represents the overall number of mutations a patient carried. Side bars represent the percentage of patients with a certain mutation. Different colors denote different types of mutation. Negative denotes the absence of any mutation. BSC, basaloid squamous cell carcinoma; KSC, keratinizing squamous cell carcinoma; NKSC, nonkeratinizing squamous cell carcinoma; SIS, squamous cell carcinoma in situ; Unknown, LUSC with unclassified subtype.
**Figure S3** The relationship between molecular and clinical features in LUAD patients. Box plot illustrating the relationship between age of the LUAD patients and *TP53* mutation. X‐axis denotes the *TP53* mutation status, negative for wild‐type. Y‐axis denotes the age of the patients in years.

**Table S1** Pathologic stage distribution of the LUAD patients according to the predominant histologic subtypes.
**Table S2** Pathologic stage distribution of the LUSC patients according to the predominant histologic subtypes.
**Table S3** Summary of concurrent and rare somatic mutations in oncogenic genes found in LUAD patients.
**Table S4** *ALK* fusions detected in 10 patients from our cohort.
