# Supplementary figures and images for: Comparative study on the mutational profile of adenocarcinoma and squamous cell carcinoma predominant histologic subtypes in Chinese non‐small cell lung cancer patients
Source: Thorac Cancer. 2019 Nov 6;11(1):103–12. doi: 10.1111/1759-7714.13208 (PMC6938761; doi:10.1111/1759-7714.13208)

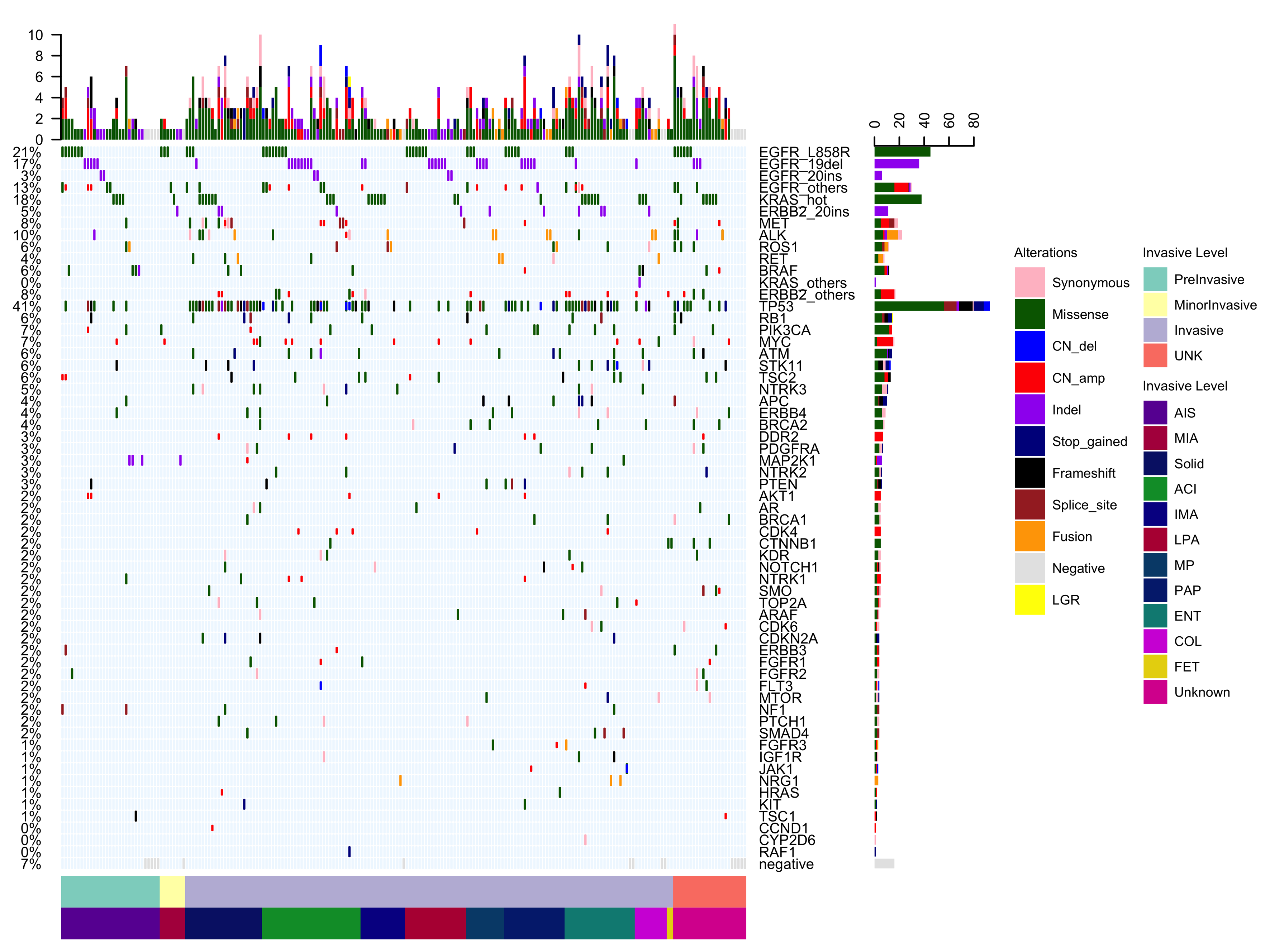

Supplement: Supplementary file 1 — Figure S1 Mutational spectrum of the LUAD patients. Each column represents a patient and each row represents a gene. Top plot represents the overall number of mutations a patient carried. Side bars represent the percentage of patients with a certain mutation. Different colors denote different types of mutation. Negative denotes the absence of any mutation. ACI, acinar adenocarcinoma; AIS, adenocarcinoma in situ; COL, colloid adenocarcinoma; ENT, enteric adenocarcinoma; FET, fetal adenocarcinoma; IMA, invasive mucinous adenocarcinoma; LPA, lepidic adenocarcinoma; MIA, minimally invasive adenocarcinoma; MP, micropapillary adenocarcinoma; PAP, papillary adenocarcinoma; Solid, solid adenocarcinoma; Unknown, LUAD with unclassified subtype. [file TCA-11-103-s001.tif]

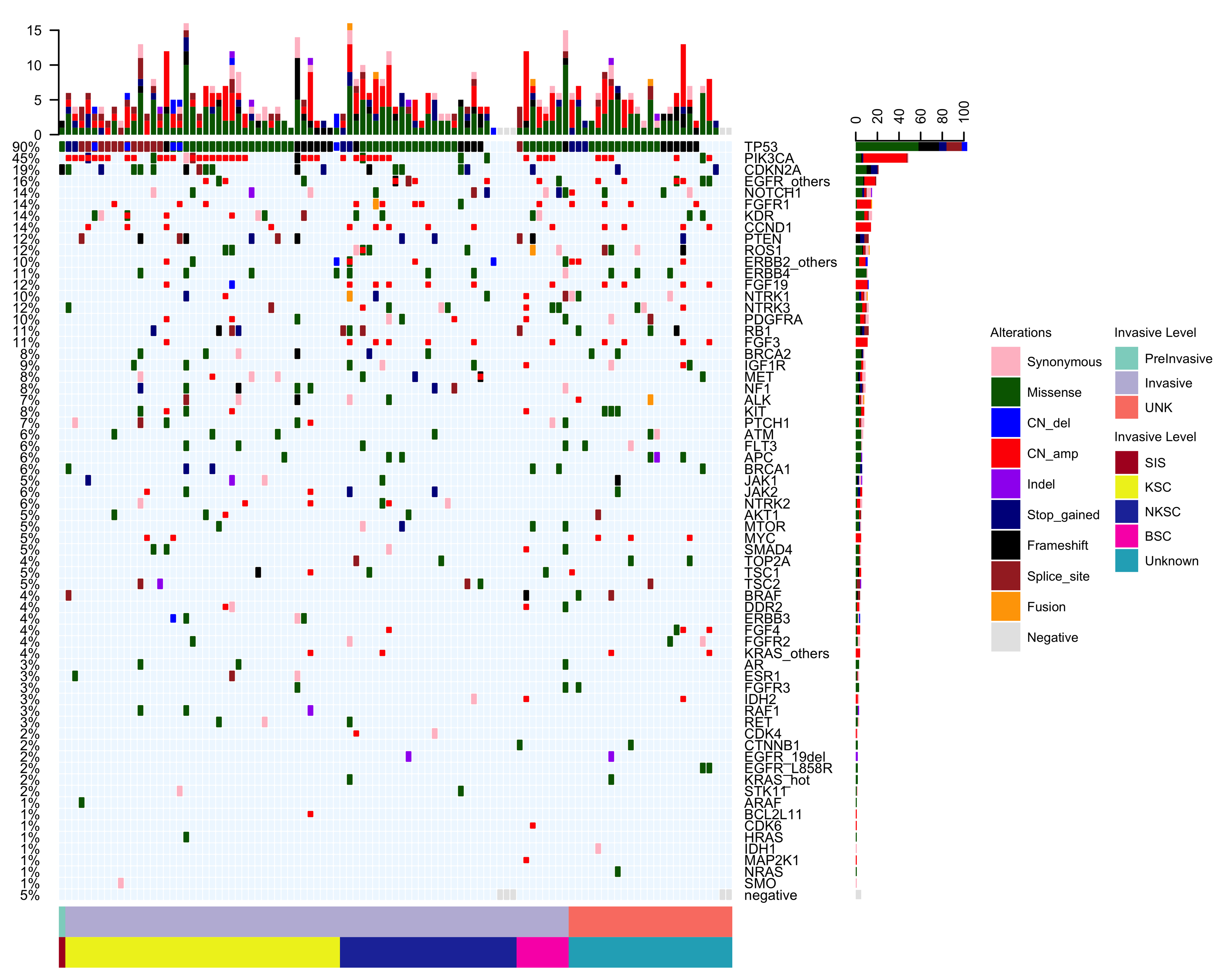

Supplement: Supplementary file 2 — Figure S2 Mutational profile of LUSC patients according to histological subtypes. Each column represents a patient and each row represents a gene. Top plot represents the overall number of mutations a patient carried. Side bars represent the percentage of patients with a certain mutation. Different colors denote different types of mutation. Negative denotes the absence of any mutation. BSC, basaloid squamous cell carcinoma; KSC, keratinizing squamous cell carcinoma; NKSC, nonkeratinizing squamous cell carcinoma; SIS, squamous cell carcinoma in situ; Unknown, LUSC with unclassified subtype. Figure S3 The relationship between molecular and clinical features in LUAD patients. Box plot illustrating the relationship between age of the LUAD patients and TP53 mutation. X‐axis denotes the TP53 mutation status, negative for wild‐type. Y‐axis denotes the age of the patients in years. [file TCA-11-103-s002.tif]
