# Supplementary material for: Comparative study on the mutational profile of adenocarcinoma and squamous cell carcinoma predominant histologic subtypes in Chinese non‐small cell lung cancer patients
Source: Thorac Cancer. 2019 Nov 6;11(1):103–12. doi: 10.1111/1759-7714.13208 (PMC6938761; doi:10.1111/1759-7714.13208)
Supplement: Supplementary file 3 — Table S1 Pathologic stage distribution of the LUAD patients according to the predominant histologic subtypes. Table S2 Pathologic stage distribution of the LUSC patients according to the predominant histologic subtypes. Table S3 Summary of concurrent and rare somatic mutations in oncogenic genes found in LUAD patients. Table S4 ALK fusions detected in 10 patients from our cohort. [file TCA-11-103-s003.docx]

Table S1. Pathologic stage distribution of the LUAD patients according to the predominant histologic subtypes

| Predominant LUAD Subtype | Total  (n=215) | Stage IA  (n=50) | Stage IB  (n=22) | Stage IIA  (n=6) | Stage IIB  (n=4) | Stage ⅢA  (n=24) | Stage IIIB  (n=3) | Stage IV  (n=13) | NA  (n=93) |
| --- | --- | --- | --- | --- | --- | --- | --- | --- | --- |
| Pre-invasive subtype | | | | | | | | | |
| Adenocarcinoma *in situ* | 31 (14.4%) | 1 (2.0%) | 0 | 0 | 0 | 0 | 0 | 0 | 30 (32.3%) |
| Minimally invasive subtype | | | | | | | | | |
| Minimally invasive | 8 (3.7%) | 2 (4.0%) | 0 | 0 | 0 | 0 | 0 | 0 | 6 (6.5%) |
| Invasive subtype | | | | | | | | | |
| Acinar | 31 (14.4%) | 13 (26.0%) | 3 (13.6%) | 0 | 1 (25.0%) | 3 (12.5%) | 1 (33.3%) | 1 (7.7%) | 9 (9.7%) |
| Solid | 24 (11.2%) | 3 (6.0%) | 3 (13.6%) | 0 | 1 (25.0%) | 6 (25.0%) | 1 (33.3%) | 1 (7.7%) | 9 (9.7%) |
| Enteric | 22 (10.2%) | 8 (16.0%) | 4 (18.2%) | 3 (50.0%) | 0 | 4 (16.7%) | 0 | 0 | 3 (3.2%) |
| Lepidic | 19 (8.8%) | 10 (20.0%) | 1 (4.5%) | 0 | 0 | 0 | 0 | 0 | 8 (8.6%) |
| Papillary | 19 (8.8%) | 3 (6.0%) | 4 (18.2%) | 1 (16.7%) | 0 | 2 (8.3%) | 0 | 1 (7.7%) | 8 (8.6%) |
| Invasive Mucinous | 14 (6.5%) | 2 (4.0%) | 1 (4.5%) | 0 | 1 (25.0%) | 2 (8.3%) | 1 (33.3%) | 0 | 7 (7.5%) |
| Micropapillary | 12 (5.6%) | 0 | 0 | 2 (33.3%) | 0 | 5 (20.8%) | 0 | 0 | 5 (5.4%) |
| Colloid | 10 (4.7%) | 5 (10.0%) | 3 (13.6%) | 0 | 1 (25.0%) | 1 (4.2%) | 0 | 0 | 0 |
| Fetal | 2 (0.9%) | 2 (4.0%) | 0 | 0 | 0 | 0 | 0 | 0 | 0 |
| Unknown | 23 (10.7%) | 1 (2.0%) | 3 (13.6%) | 0 | 0 | 1 (4.2%) | 0 | 10 (76.9%) | 8 (8.6%) |

Table S2. Pathologic stage distribution of the LUSC patients according to the predominant histologic subtypes

| Predominant LUSC Subtype | Total  (n=103) | Stage IA  (n=15) | Stage IB  (n=10) | Stage IIA  (n=9) | Stage IIB  (n=11) | Stage ⅢA  (n=8) | Stage IIIB  (n=3) | Stage IV  (n=3) | NA  (n=44) |
| --- | --- | --- | --- | --- | --- | --- | --- | --- | --- |
| Pre-invasive subtype | | | | | | | | | |
| Squamous cell carcinoma *in situ* | 1 (1.0%) | 0 | 0 | 0 | 0 | 0 | 0 | 1 (33.3%) | 0 |
| Invasive subtypes | | | | | | | | | |
| Basaloid | 8 (7.8%) | 1 (6.7%) | 1 (10.0%) | 3 (33.3%) | 1 (9.1%) | 0 | 0 | 0 | 2 (4.5%) |
| Keratinizing | 42 (40.8%) | 6 (40.0%) | 2 (20.0%) | 1 (11.1%) | 5 (45.5%) | 5 (62.5%) | 1 (33.3%) | 1 (33.3%) | 21 (47.7%) |
| Non-keratinizing | 27 (26.2%) | 4 (26.7%) | 3 (30.0%) | 3 (33.3%) | 4 (36.4%) | 1 (12.5%) | 0 | 0 | 12 (27.3%) |
| Unknown | 25 (24.3%) | 4 (26.7%) | 4 (40.0%) | 2 (22.2%) | 1 (9.1%) | 2 (25.0%) | 2 (66.7%) | 1 (33.3%) | 9 (20.5%) |

Table S3. Summary of concurrent and rare somatic mutations in oncogenic genes found in LUAD patients

| Patient ID | Gene | Mutation type | Somatic mutation | Allele frequency |
| --- | --- | --- | --- | --- |
| 2 concurrent mutations in a single known oncogenic gene | | | | |
| AH0053 | *EGFR* | missense variant | **NM_005228.3(EGFR):c.2303G>T(p.Ser768Ile) *** | 11.7% |
|  | *EGFR* | missense variant | **NM_005228.3(EGFR):c.2512C>G(p.Leu838Val) *** | 12.6% |
| RS1908 | *KRAS* | missense variant | NM_033360.3(KRAS):c.37G>T(p.Gly13Cys) | 29.04% |
|  | *KRAS* | missense variant | NM_033360.3(KRAS):c.34G>T(p.Gly12Cys) | 28.84% |
| AL0050 | *RET* | fusion | CCDC6-RET(C1:R12) | 33% |
|  | *RET* | fusion | **CCSER2-RET(C3:R12) *** | 31.7% |
| 2 concurrent mutations in 2 different known oncogenic genes | | | | |
| AA0399 | *EGFR* | missense variant | NM_005228.3(EGFR):c.2573T>G(p.Leu858Arg) | 33% |
|  | *KRAS* | missense variant | NM_033360.3(KRAS):c.183A>C(p.Gln61His) | 38.5% |
| AH0017 | *RB1* | stop gained | NM_000321.2(RB1):c.2488A>T(p.Arg830*) | 15.5% |
|  | *TP53* | stop gained | NM_000546.5(TP53):c.892G>T(p.Glu298*) | 19.5% |
| AH0039 | *EGFR* | missense variant | NM_005228.3(EGFR):c.2156G>C(p.Gly719Ala) | 36.2% |
|  | *MET* | copy number amplification |  | 3.48 |
| AL0006 | *EGFR* | missense variant | NM_005228.3(EGFR):c.2573T>G(p.Leu858Arg) | 34.5% |
|  | *ERBB2* | missense variant | NM_004448.3(ERBB2):c.929C>T(p.Ser310Phe) | 31.1% |
| AT0030 | *EGFR* | missense variant | NM_005228.3(EGFR):c.2573T>G(p.Leu858Arg) | 9.6% |
|  | *ERBB2* | missense variant | NM_004448.3(ERBB2):c.929C>T(p.Ser310Phe) | 11.1% |
| AH0034 | *EGFR* | missense variant | NM_005228.3(EGFR):c.2573T>G(p.Leu858Arg) | 41.3% |
|  | *ERBB2* | copy number amplification |  | 4.43 |
| AT0053 | *EGFR* | missense variant | NM_005228.3(EGFR):c.2573T>G(p.Leu858Arg) | 32.7% |
|  | *ERBB2* | copy number amplification |  | 3.95 |

Table S3 continued

| Patient ID | Gene | Mutation Type | Somatic mutation | Allele frequency |
| --- | --- | --- | --- | --- |
| 3 concurrent mutations in 2 or 3 known oncogenic genes | | | | |
| RS2501 | *ERBB2* | copy number amplification |  | 2.82 |
|  | *FGFR3* | fusion | FGFR3-TACC3(F17:T8) | 18.02% |
|  | *EGFR* | missense variant | NM_005228.3(EGFR):c.2573T>G(p.Leu858Arg) | 17.68% |
| RS2520 | *ERBB2* | copy number amplification |  | 3.03 |
|  | *EGFR* | disruptive inframe deletion | NM_005228.3(EGFR):c.2235_2249del(p.Glu746_Ala750del) | 68.45% |
|  | *EGFR* | copy number amplification |  | 7.04 |
| single rare mutation in known oncogenic gene | | | | |
| AT0054 | *NRG1* | fusion | CD74-NRG1(C6:N2) | 8.6% |
| RS2502 | *NRG1* | fusion | CD74-NRG1(C6:N2) | 18.1% |
| AL0063 | *NRG1* | fusion | CD74-NRG1(C7:N2) | 11.7% |
| RS2503 | *RB1* | large genomic rearrangement | NM_000321.2(RB1):exon25-27cn_del | 80% |
| AL0038 | *BRAF* | disruptive inframe insertion | **NM_004333.4(BRAF):c.1509_1517dupAGTACTCAG(p.Arg506_Lys507insValLeuArg) *** | 12.8% |

* Somatic mutations in bold are rare mutations

Table S4. *ALK* fusions detected in 10 patients from our cohort

| Patient ID | Histologic subtype | *ALK* fusion partner genes | Variant fusion position | Allele Frequency |
| --- | --- | --- | --- | --- |
| AI0070 | LUSC | *EML4* | Intron 13; Exon 20 | 1.30% |
| AA0306 | LUAD | *EML4* | Intron 6; Intron 19 | 73.30% |
| RS2508 | LUAD | *EML4* | Intron 6; Intron 19 | 10.39% |
| AT0045 | LUAD | *EML4* | Intron 20; Intron 19 | 40.00% |
| RS2509 | LUAD | *EML4* | Intron 20; Intron 19 | 7.06% |
| AL0057 | LUAD | ***ERBB4 **** | Intron 19; Intron 19 | 26.20% |
| AH0051 | LUAD | ***PRR20A **** | intergenic; Intron 19 | 33.40% |
|  |  | ***RHOB **** | intergenic; Intron 19 | 28.20% |
| AL0060 | LUAD | *EML4* | Intron 13; Intron 19 | 28.30% |
|  |  | ***EXOC6B **** | Intron 18; Intron 19 | 26.00% |
| AL0078 | LUAD | *EML4* | Intron 6; Intron 19 | 31.80% |
|  |  | ***TTN **** | Intron 38; Intron 19 | 25.00% |
| AI0049 | LUAD | *EML4* | Intron 6; Intron 19 | 13.90% |
|  |  | ***ACVR1 **** | Intron 9; Intron 19 | 17.40% |
|  |  | ***TACR1 **** | intergenic; Exon 19 | 25.50% |

* genes in **bold** are unreported *ALK* fusion partner genes
